# Total clavicle reconstruction with free peroneal graft for the surgical management of chronic nonbacterial osteomyelitis of the clavicle: a case report

**DOI:** 10.1186/s12891-019-2588-y

**Published:** 2019-05-13

**Authors:** Patrick Goetti, Chau Pham, Nicolas Gallusser, Fabio Becce, Pietro G. di Summa, Frédéric Vauclair, Stéphane Cherix

**Affiliations:** 1 Department of Orthopaedics and Traumatology, Lausanne University Hospital and University of Lausanne, Rue du Bugnon 46, 1011 Lausanne, Switzerland; 2 Department of Diagnostic and Interventional Radiology, Lausanne University Hospital and University of Lausanne, Lausanne, Switzerland; 3 Department of Plastic, Reconstructive and Hand Surgery, Lausanne University Hospital and University of Lausanne, Lausanne, Switzerland

**Keywords:** Chronic nonbacterial osteomyelitis, Claviclectomy, Clavicle reconstruction, Free peroneal graft, SAPHO syndrome

## Abstract

**Background:**

Chronic nonbacterial osteomyelitis (CNO) is a rare chronic autoinflammatory syndrome affecting mainly children and young adults. The natural history of the disease is marked by recurrent pain as the mainstay of inflammatory outbreaks. Typical radiographic findings are osteosclerosis and hyperostosis of the medial clavicle, sternum and first rib. Compression of the brachial plexus is exceedingly rare and one of the few surgical indications. Literature on total clavicle reconstruction is scarce. While claviclectomy alone has been associated with fair functional and cosmetic outcomes, several reconstruction techniques with autograft, allograft or even cement (“Oklahoma prosthesis”) have been reported with the aim of achieving better pain control, cosmetic outcome and protecting the brachial plexus and subclavian vessels. We herewith report a unique case of complicated CNO of the clavicle treated with total clavicle reconstruction using a free peroneal graft.

**Case presentation:**

A 21-year-old female patient presented with CNO of her left clavicle, associated with recurrent, progressive and debilitating pain as well as limited range of motion. In recent years, she started complaining of paresthesia, weakness and pain radiating to her left arm during arm abduction. The clavicle diameter reached 6 cm on computed tomography, with direct compression of the brachial plexus and subclavian vessels. Following surgical biopsy for diagnosis confirmation, she further developed a chronic cutaneous fistula. Therefore, a two-stage total clavicle reconstruction using a vascularized peroneal graft stabilized by ligamentous reconstruction was performed. At two-year follow-up, complete pain relief and improvement of her left shoulder Constant-Murley score were observed, along with satisfactory cosmetic outcome.

**Conclusions:**

This case illustrates a rarely described complication of CNO with direct compression of the brachial plexus and subclavian vessels, and chronic cutaneous fistula. To our knowledge, there is no consensus regarding the optimal management of this rare condition in this context. Advantages and complications of clavicle reconstruction should be carefully discussed with patients due to limited evidence of superior clinical outcome and potential local and donor-site complications. While in our case the outcomes met the patient’s satisfaction, it remains an isolated case and further reports are awaited to help surgeons and patients in their decision process.

## Background

Chronic nonbacterial osteomyelitis (CNO) is a rare chronic autoinflammatory syndrome affecting mainly children and young adults. Its true incidence might actually be underestimated [1]. When associated with acne, synovitis and pustulosis, CNO is reported as SAPHO syndrome [2]. Clinical presentation may vary widely as heat and/or swelling of the affected bone segment are not always present, which makes the diagnosis challenging [3]. Cortical bone thickening with narrowing of the medullary canal on plain radiography are suggestive imaging findings [4]. However, histopathological confirmation is mandatory to rule out neoplasia [5]. These factors may explain why the diagnosis is often delayed, leading to progressive limitation in range of motion (ROM) due to recurrent pain. Treatment is mainly conservative, with pain control using nonsteroidal anti-inflammatory drugs (NSAID) during recurrent inflammatory outbreaks. Corticosteroids, colchicine and fenestration combined with bone curettage have been proposed as alternative treatment options. While bone resection is not indicated as a primary therapeutic procedure, it may become necessary when dealing with late-stage complications of an extensive disease such as debilitating pain and/or local mass effect on adjacent neurovascular structures [6].

We herewith report a unique case of complicated CNO of the clavicle treated with a two-stage total clavicle reconstruction using a free vascularized peroneal graft stabilized by ligamentous reconstruction.

## Case presentation

A 21-year-old Caucasian female patient presented to our consultation with CNO of her left clavicle. The associated pain was recurrent, progressive and eventually led to work stoppage as a secretary. Symptoms started at the age of nine, with initial plain radiographs showing fusiform sclerotic bone remodeling of the medial third of the left clavicle. There was no further relevant past medical history. Percutaneous bone biopsy at that time revealed fragments of immature woven bone, with no signs of malignancy. Histopathological and microbiological analyses excluded etiologies such as infection, neoplasia, vascular or metabolic disorders, and the diagnosis of CNO was retained. She was thus treated conservatively with NSAID.

**Fig. 1:**
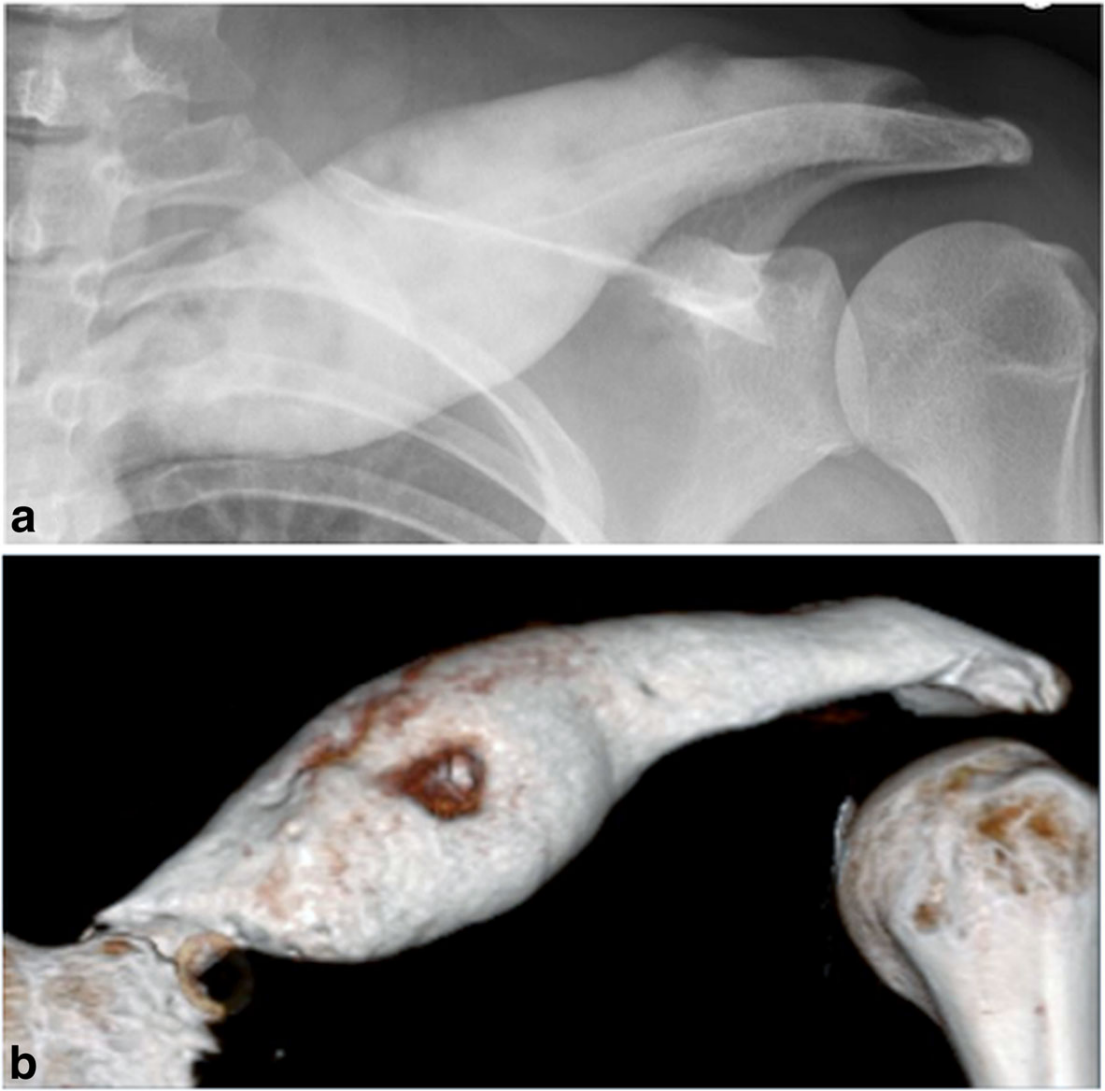
**a** Anteroposterior radiograph and **b** volume-rendered computed tomography image of the left clavicle prior to surgical reconstruction

Over the years, she started complaining of paresthesia, weakness and pain radiating to her left arm on abduction or elevation of her left shoulder. Clavicle diameter reached 6 cm on computed tomography. Lateral progression of osteosclerosis was noted, with involvement of the acromioclavicular joint and compression of the brachial plexus and subclavian vessels (Fig. 1). Malignant transformation was excluded by a further surgical bone biopsy at the age of 20, but wound dehiscence and chronic cutaneous fistula developed a few months later.

**Fig. 2:**
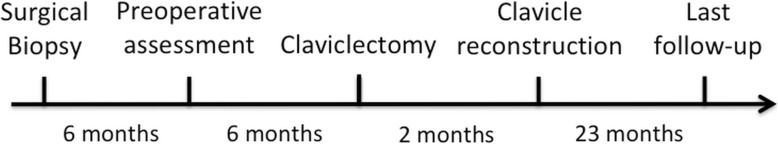
Timeline of interventions

**Fig. 3:**
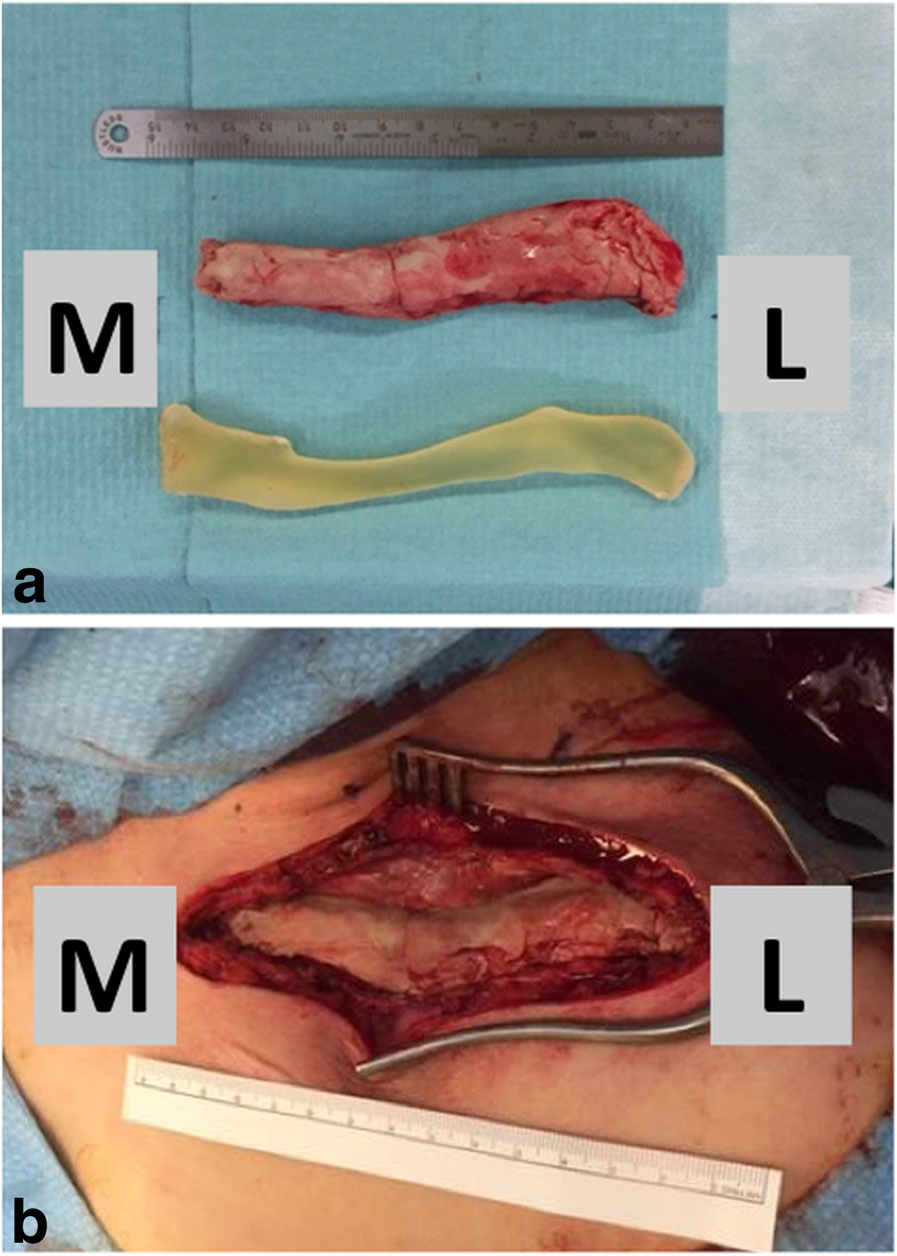
First stage: **a** contralateral clavicle model (bottom) with custom-molded antibiotic-loaded cement spacer (top), **b** antibiotic-loaded cement spacer in place after clavicle excision L = lateral, M = medial

At the time the patient (aged 21) was referred to our institution, she had a 5/10 pain score at rest using the visual analogue scale (VAS). The shoulder ROM was limited to 90° of flexion and elevation, while internal and external rotation were preserved. The Constant-Murley shoulder outcome score was at 35/100 (this score evaluates pain, activities of daily living, strength and shoulder ROM [7]). We then proposed and performed a two-stage reconstruction of the left clavicle (Fig. 2). Following claviclectomy, the defect was filled with a custom-made antibiotics-loaded cement spacer, allowing the formation of an induced membrane according to the Masquelet technique, while total clavicle reconstruction using a free vascularized peroneal graft was performed 8 weeks after the index procedure (Fig. 3).

**Fig. 4:**
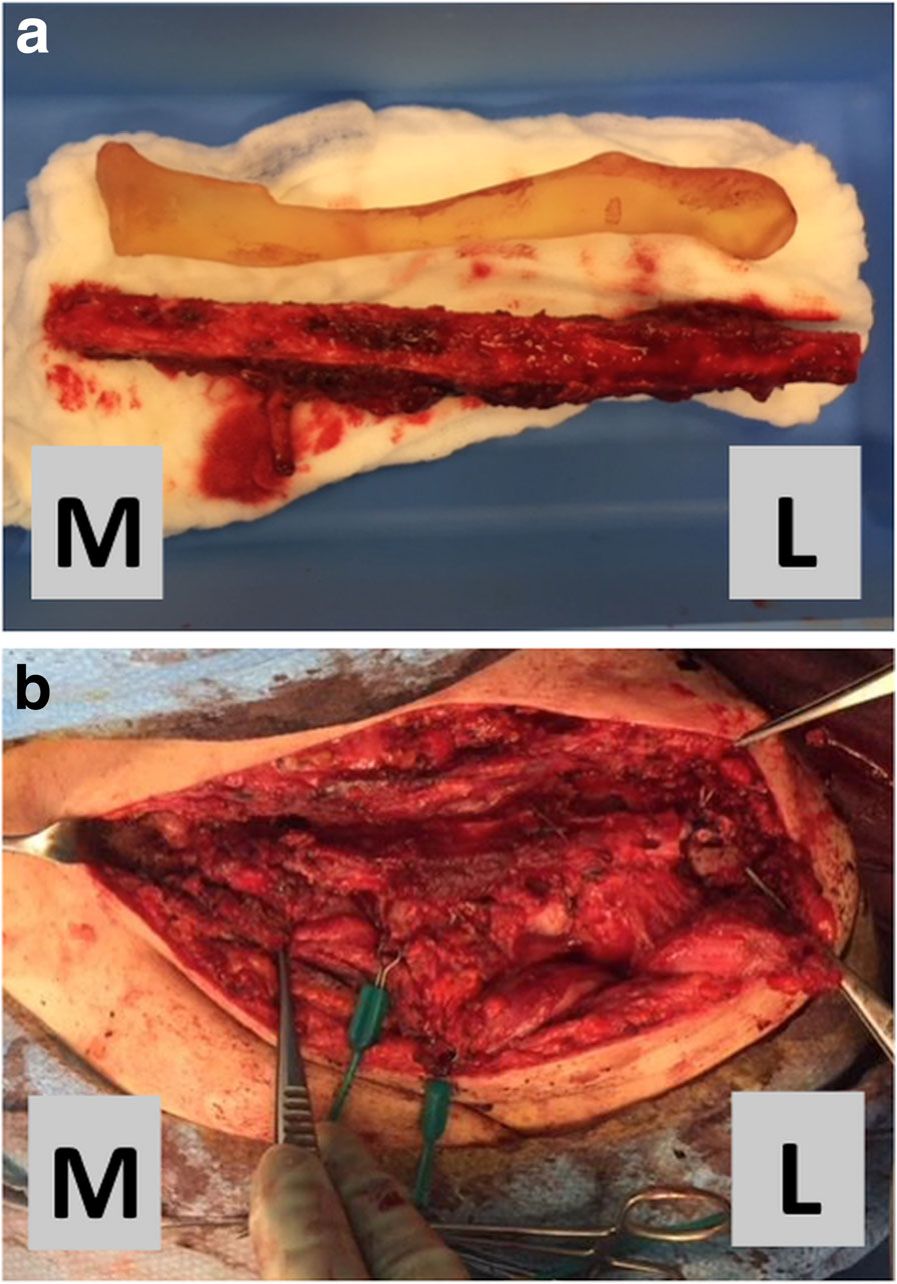
Second stage: **a** contralateral clavicle model (top) with harvested free peroneal graft (bottom), **b** free peroneal graft in place, stabilized with hamstring tendon reconstruction of sterno- and acromioclavicular joints reinforced with suture anchors L = lateral, M = medial

**Fig. 5:**
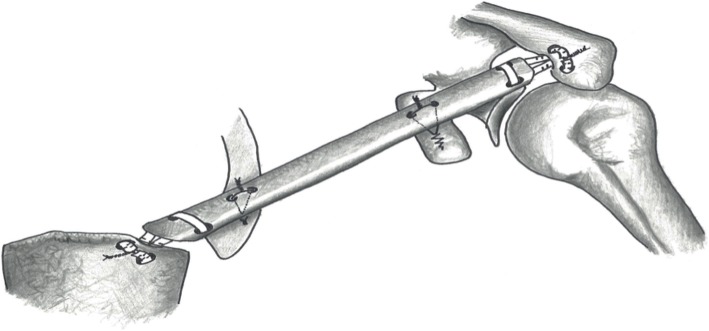
Graphic illustration of the hamstring tendons reconstruction of the sterno- and acromioclavicular joints using bony tunnels. Stabilization was reinforced with suture anchors placed at the base of the coracoid and on the antero-superior aspect of the first rib

**Fig. 6:**
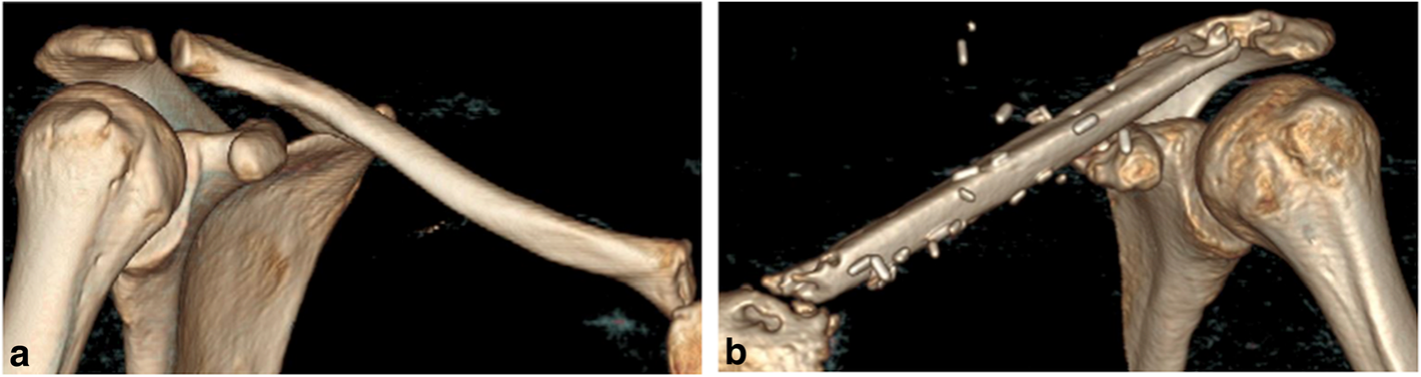
Volume-rendered computed tomography images of **a** right and **b** left clavicles after surgical reconstruction of the left clavicle

**Fig. 7:**
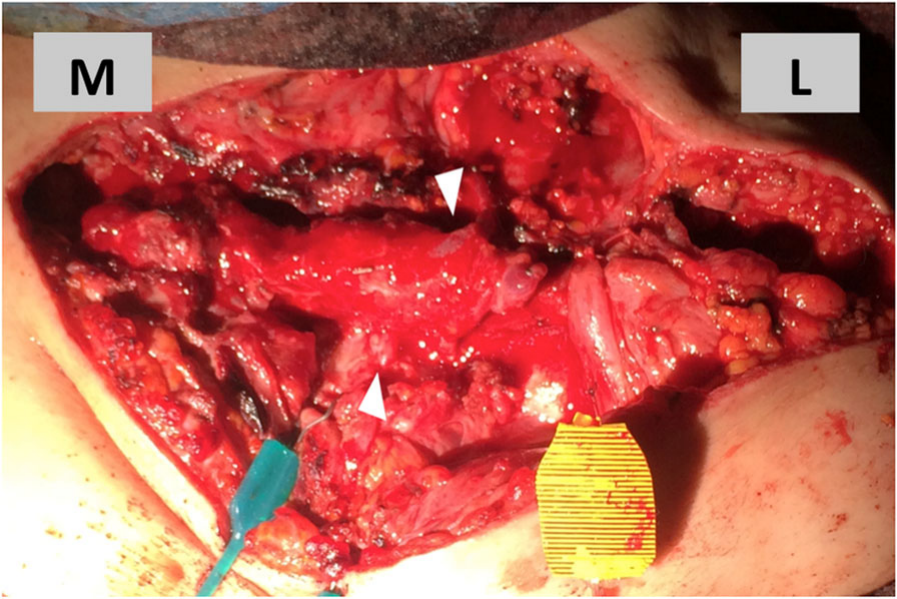
Vascular bundle of the free peroneal graft anastomotized to a branch of the external jugular vein (upper arrowhead) and the thoracoacromial artery (lower arrowhead) L = lateral, M = medial

For surgical reconstruction, a 3D-printed model based on the healthy contralateral right clavicle was used to conform the new left clavicle. After harvest, the free fibula was positioned in the Masquelet cavity (Fig. 4). All four medial and lateral anchorings of the clavicle were reconstructed. Hamstring tendons were passed through bone tunnels in the sterno- and acromioclavicular joints. Tension in anchorings was low to allow for free shoulder elevation and antepulsion up to 90°. Stabilization was reinforced with suture anchors placed at the base of the coracoid and on the antero-superior aspect of the first rib (Figs. 5 and 6). Finally, the vascular bundle was anastomotized to the thoracoacromial artery and a branch of the external jugular vein (Fig. 7).

Perifistular cultures revealed usual skin pathogens. Macroscopically, we observed no signs of suppurative infection or sequestration. Histologically, there were intratrabecular spaces filled with loose fibrovascular tissue containing inflammatory cells (mainly lymphocytes, plasma cells, macrophages and a few polymorphonuclear cells). Periodic acid-Schiff, Grocott, Gram and Ziehl-Neelsen colorations were all negative. We found no evidence of organisms, eosinophils, granulomatous foci, abscess formations, sequestrae, and signs of malignancy or Paget’s disease. Clinical presentation, imaging findings and histopathological analyses led to the final diagnosis of CNO.

Postoperative evolution was favorable with eventless wound healing. The left shoulder was immobilized for 6 weeks, then progressive active and passive motion were started. Follow-up consultation at 23 months showed satisfactory cosmetic and functional outcomes. Left shoulder ROM returned to the level of preoperative assessment 3 months after surgical reconstruction. While pain VAS was 2/10 at 3 months, it was 0/10 at last follow-up (23 months) without pain medication. The evolution of the Constant-Murley shoulder score was satisfactory with an improvement to 68 points at 2 years. The long-term evolution met the patient’s satisfaction, which was evaluated at 8 on a 10-point rating scale.

## Discussion and conclusions

There is currently limited evidence on the optimal management of CNO, with only few small case series and case reports in the literature. Evidence concerning segmental bone excision and reconstruction is even scarcer and, to our knowledge, limited to a single case report where the authors opted to use a bone transport technique with external fixation [6].

The complex anatomy of the clavicle is characterized by the strong sternoclavicular joint capsule and costoclavicular ligaments on the medial aspect and the coracoclavicular and acromioclavicular ligaments laterally [8]. Therefore, the clavicle not only represents a link between the trunk and the shoulder but also contributes to shoulder motion and strength by transmitting the forces of the trapezius muscle to the scapula [9]. In the case of a claviclectomy, protraction and retraction of the scapula is increased, leading to secondary weakness in overhead activities [10]. Furthermore, the clavicle also acts as a shield to protect the underlying neurovascular structures, which are located just beneath its medial third [11]. Finally, esthetics has also been pointed out as a potential concern after total or partial claviclectomy, particularly in female patients [12].

Several case reports of total claviclectomy reported satisfactory results in terms of pain control and shoulder function. However, associated complications such as infections and vascular lesions should not be overlooked [13–15]. These observations were further questioned by other reports showing less satisfactory outcomes in terms of pain control, ROM, strength and patients’ satisfaction [16, 17]. A recent systematic review did not suggest any evidence of better clinical outcome after surgical reconstruction of the clavicle [18]. Various clavicle reconstruction techniques have been proposed in the literature (mainly segmentary and mostly in cases of nonunion or reconstruction after tumor excision), including autograft, allograft and synthetic materials. Autografts have been described in the form of free fibula or “Eve procedure” with a costal graft [19–21]. The main advantage of this procedure is to bring healthy vascularized tissue and improve the long-term outcome by enhancing the healing of the surrounding soft tissues. On the other hand, fibula harvesting may be associated with infection, fracture and knee or ankle instability at the donor site.

In our case, the indication to perform a total claviclectomy was dictated by the occurrence of an infectious complication with development of a chronic cutaneous fistula, as well as by pain and neurologic complaints due to local mass effect. We thus opted to reconstruct the clavicle based on the patient’s young age, the good long-term evolution of the disease and the aim for both an esthetic and functional surgical reconstruction. We opted for a two-stage approach to total clavicle reconstruction, with a custom-made antibiotics-loaded cement spacer. This option had two main advantages. First, the infectious background was in our opinion not suitable for a single-stage procedure and we further recommend this approach in case of doubtful diagnosis. The second argument was that the cement spacer would favor stability and biological integration of the vascularized peroneal graft. We used contralateral computed tomography images and preferred to maintain clavicle length rather than its proper anatomical shape for our surgical reconstruction. While clavicular length has been linked with improved functional outcome in clavicle fractures, there is limited evidence concerning the importance of its shape. Furthermore, this choice spared osteotomy of the peroneal graft and thus eliminated the risk of nonunion and symptomatic metal hardware [22, 23]. The improvement in the Constant-Murley score noted in our case might indicate that clavicular length is sufficient to improve or maintain shoulder function after total clavicle excision, even though the ROM returned to its preoperative level but did not surpass it.

We opted to fix our peroneal graft using an anatomic ligamentous reconstruction. To the best of our knowledge, the association of a free vascularized bone graft with ligamentous reconstruction has not been described so far. When dealing with total clavicle reconstruction, this combination makes even more sense. It not only avoids any need for subsequent surgery to remove symptomatic or infected foreign materials, but the biologic fixation also ensures a long-lasting stability of the reconstruction. Another key point in this case was the combination of a free flap with the concept of induced biological membrane [24]. While not mandatory, this might facilitate integration in a compromised environment.

Our technique showed an effective and persistent pain remission associated with a functional improvement at two years follow-up. The closest report to ours is the one by Kalbermatten et al., with a case of late clavicular reconstruction using a free peroneal graft 23 years after the index procedure [25]. They used an osteotomy guide to reconstruct the S-shaped clavicle, which was fixed with a plate and screws to maintain its alignment as well as Kirchner wires and suture anchors to attach it to the acromion and sternum. Besides a modest improvement of 10° in shoulder ROM in the sagittal plane, the procedure was reported as effective on pain control, which was unfortunately not assessed using VAS. On the other hand, Lin et al. reported results in five oncologic patients with subtotal (instead of total) claviclectomy using a cement prosthesis to replace the clavicle [26]. Any direct comparison with this study should thus be made with caution. The authors also used VAS pain score and found significant improvements from 8.0 (range, 6–9) to 1.4 (range, 1–2). They further reported functional outcome using the American Shoulder and Elbow Surgeons shoulder outcome score, which improved from 42.4 ± 9.0 to 92.4 ± 3.3. While this score does not directly assess strength, it is comparable to the Constant-Murley score. The long-term outcome of this technique is not yet known, this method having mainly been used in patients with limited life-time expectancy. Finally, Li et al. reviewed 6 clavicle reconstructions using massive allografts, all of them performed after subtotal claviclectomy for malignancies [27]. In their retrospective study, the mean postoperative Constant-Murley score was 85. However, it was associated with a high complication rate with two allografts secondarily removed due to infection and nonunion.

In conclusion, advanced complicated CNO is a rare indication for claviclectomy. This implies that advantages and complications of clavicle reconstruction should be carefully discussed with patients due to limited evidence of superior clinical outcome and potential local and donor-site complications. While in our case the outcome met the patient’s satisfaction, it remains an isolated case and further reports are awaited to help surgeons and patients in their decision process.

## Abbreviations

CNO: Chronic nonbacterial osteomyelitis
NSAID: Nonsteroidal anti-inflammatory drugs
ROM: Range of motion
SAPHO: Synovitis acne pustulosis hyperostosis osteitis
VAS: Visual analogue scale
